# The Pediatric Recruitment Engine: A Transparent Computational Model for Inspecting Assumptions During Positive End-Expiratory Pressure Titration

**DOI:** 10.7759/cureus.117389

**Published:** 2026-10-03

**Authors:** Paolo Taffache

**Affiliations:** 1 General and Pediatric Anesthesia and Intensive Care Unit, Istituto di Ricovero e Cura a Carattere Scientifico (IRCCS), Azienda Ospedaliero-Universitaria di Bologna Policlinico di Sant’Orsola, Bologna, ITA

**Keywords:** computational model, lung volume recruitment, mechanical ventilation, medical education technology, pediatric mechanical ventilation, positive end-expiratory pressure (peep)

## Abstract

Positive end-expiratory pressure (PEEP) can reduce lung collapse, while higher pressures may cause overdistension and unfavorable dead space or pressure effects. The Pediatric Recruitment Engine (PRE) v0.7 is an open-source computational model for supervised teaching about these competing assumptions. It represents 2,560 heterogeneous virtual lung units in 16 conceptual regions. A fixed sequence of increasing and decreasing PEEP updates each unit's probability of remaining open and produces model-derived indices of recruitment, collapse, mechanics, shunt, oxygenation, dead space, carbon dioxide clearance, and regional balance. In one seeded scenario for a 2-year-old, 12-kg child under the moderate impairment preset, several balance and clearance indices selected 16 cmH2O, while the intentionally monotonic oxygenation surrogate selected the upper tested value of 24 cmH2O. Re-running the frozen code reproduced its archived numerical outputs, and 10 scripted execution and expected-behavior checks passed. These checks do not establish independent code verification or physiologic validity. A focused exploratory sensitivity analysis showed that some selected indices changed when illustrative model assumptions were varied. PRE uses no patient-level data, has not been calibrated or externally validated, and cannot recommend patient-specific PEEP. Its code and archived release are publicly available; the full mathematical specification and new exploratory analyses accompany this revision.

## Introduction

Positive end-expiratory pressure (PEEP) is the pressure left in the airways at the end of exhalation. It can help keep lung units open and support gas exchange, but too little pressure may allow collapse while too much may overdistend already open regions. Pediatric acute respiratory distress syndrome (ARDS) guidance therefore recommends considering several physiologic signals and possible harms when adjusting PEEP, rather than pursuing one universal target [1,2].

These trade-offs matter in children because lung volume, airway closure, and the distribution of ventilation vary with age and anesthesia [3-5]. Lung ultrasound can show changes in aeration and atelectasis. Electrical impedance tomography (EIT), a bedside method for observing regional ventilation, can help characterize collapse and overdistension during PEEP changes [6-8]. Neither a change in oxygenation nor one imaging signal captures every effect of a pressure change.

Conventional teaching uses physiologic principles, bedside measurements, and clinical cases to explain PEEP titration. A transparent model can complement these approaches by letting learners change an explicit assumption and see how model-derived outputs respond. PRE is intended to expose these dependencies for discussion, not to reproduce a child's physiology or choose a ventilator setting.

A small computational model can make these competing effects easier to discuss. PRE v0.7 represents them as explicit assumptions that a learner can inspect, rather than as measurements from a child. Smooth curves and apparent optima may otherwise create false confidence. This technical report describes the frozen software, its workflow, scripted checks, one reproducible worked example, an exploratory assumption-sensitivity analysis, and the limits of interpretation.

## Technical report

Purpose and software scope

PRE v0.7 is written in Python and released under the Apache 2.0 license. The public repository contains the engine, runner script, dependency list, archived outputs, figures, and earlier technical documentation [9]. The software is for supervised education and research into model assumptions only.

PRE does not receive bedside data. Age, weight, tidal volume, inspired oxygen fraction, an impairment preset, and protocol settings define an illustrative computational scenario. Outputs named oxygenation, saturation, shunt, dead space, carbon dioxide clearance, driving pressure, and plateau pressure are mathematical surrogates: numbers produced by chosen equations. They are not predictions of partial pressure of oxygen in arterial blood (PaO2), oxygen saturation (SpO2), partial pressure of carbon dioxide in arterial blood (PaCO2), end-tidal carbon dioxide (EtCO2), measured mechanics, regional strain, or clinical outcome.

Model architecture

The default virtual lung has 16 regions on a 4-by-4 conceptual grid, with 160 units per region (2,560 units in total). The grid is a teaching visualization, not an EIT reconstruction. Units in more dependent regions receive higher opening-pressure shifts and greater perfusion weights. Each virtual unit has a sampled opening pressure, a lower closing pressure, a compliance coefficient, perfusion and ventilation weights, and an overdistension threshold. A fixed random seed makes a given scenario reproducible.

Each unit has an opening probability rather than a simple open-or-closed label. During increasing PEEP, that probability rises near the assigned opening pressure. During decreasing PEEP, units close gradually below their closing pressures. Thus the model can give different recruitment values at the same PEEP depending on the preceding pressure sequence; this is the built-in hysteresis. A separate rule increases the overdistension probability above a unit-specific threshold. The healthy-like, mild, moderate, and severe presets are conceptual test settings, not calibrated clinical phenotypes.

Mean opening probabilities give the recruitment and collapse indices. The compliance surrogate adds contributions from open units and applies an overdistension penalty. Perfusion assigned to less-open units contributes to a shunt surrogate, from which the oxygenation surrogate is calculated. A dead-space surrogate increases with overdistension and is a simple measure of ventilation maldistribution. The carbon dioxide-clearance surrogate then combines compliance, dead-space burden, and a pressure penalty. Regional and global balance indices combine selected terms. The coefficients were chosen to make qualitative relationships inspectable; none was fitted to pediatric data.

The full equations, sampling distributions, coefficient values, regional definitions, target-selection rules, and assumptions are specified in the Appendices. The equations document the frozen software; they are not empirically fitted pediatric relationships.

Simulation protocol and internal checks

The worked example uses a 2-year-old, 12-kg child, the moderate impairment preset, tidal volume 6 mL/kg, inspired oxygen fraction 0.60, 160 units per region, seed 111, and an overdistension penalty weight of 1.65. The programmed sequence increases PEEP from 0 to 24 cmH2O and then decreases it from 24 to 2 cmH2O, in 2-cmH2O steps. This broad range is a software stress test and is not a ventilator recommendation.

The frozen runner includes 10 scripted checks. Check T01 detects missing numeric scalar outputs. Checks T02-T10 compare the output with expected relationships in predefined scenarios, including pressure-related direction, hysteresis, ordering of selected indices, and ordering across conceptual presets. Many of these relationships follow directly from the equations and scenario settings. A separate rerun compared regenerated numerical outputs with archived files. No independent reference implementation, numerical accuracy benchmark, pediatric dataset, or external physiologic validation was used.

Worked model behavior

A clean rerun of the frozen scripts reproduced the archived numerical values. On the ascending limb, the model-derived recruited fraction increased from 0.000 at 0 cmH2O to 0.994 at 24 cmH2O; the overdistended fraction increased from 0.000 to 0.313. At 12 cmH2O, the recruited fraction on the descending limb exceeded that on the ascending limb by 0.591. Figure 1 displays this hysteresis, which was deliberately built into the update rule.

**Figure 1:**
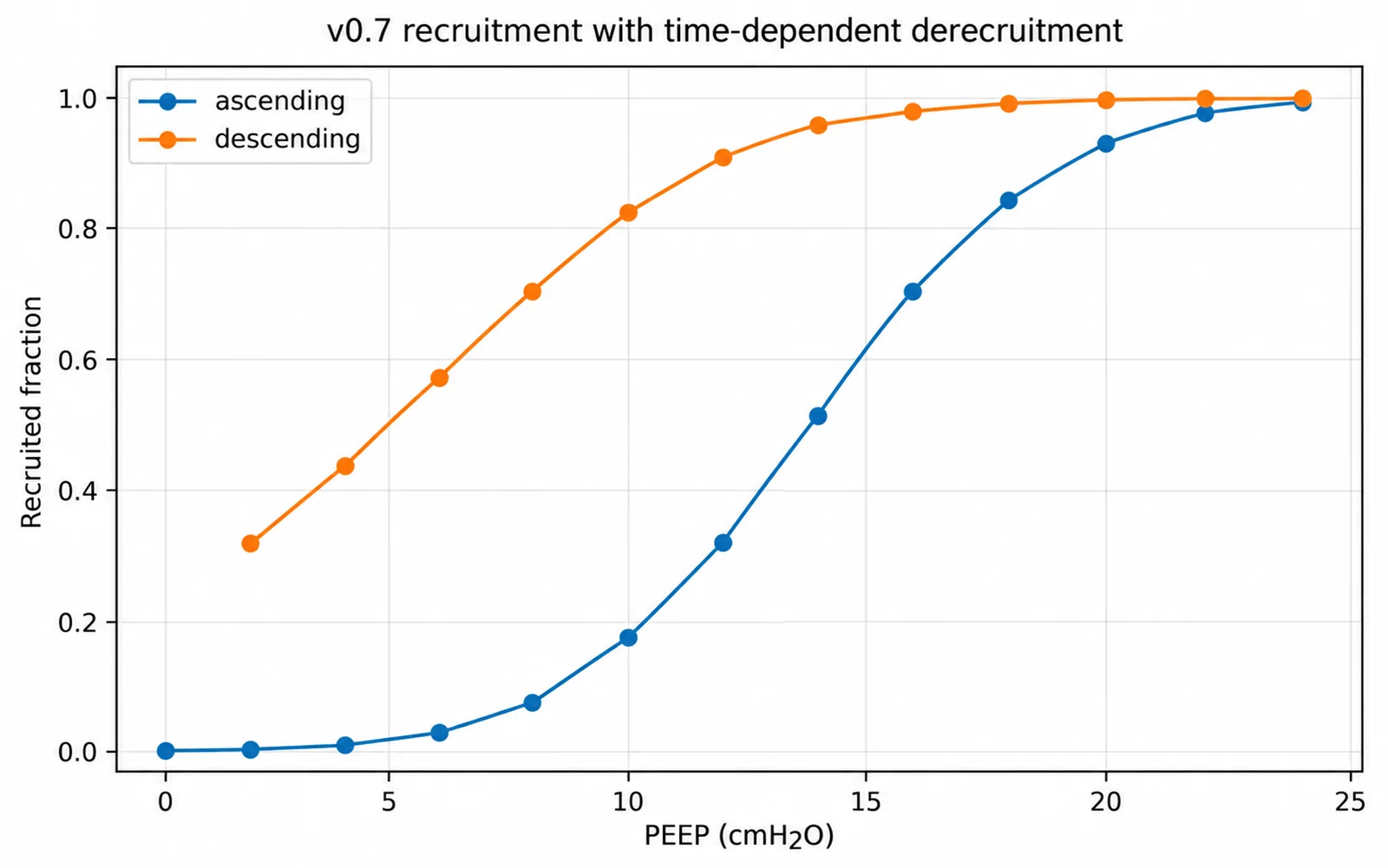
Recruitment hysteresis in the Pediatric Recruitment Engine Recruitment hysteresis produced by the sequential derecruitment rule in Pediatric Recruitment Engine (PRE) v0.7. The descending limb remains more recruited than the ascending limb at matched positive end-expiratory pressure (PEEP) values because state depends on the preceding pressure sequence. The term "time-dependent" in the plotted title refers to dependence on sequential simulation steps and not to a calibrated physical time scale. The curve is a model output and is not calibrated to pediatric recruitment or derecruitment kinetics.

On the descending limb, the regional balance, global balance, compliance, minimum driving pressure, carbon dioxide clearance, minimum carbon dioxide retention risk, and composite gas exchange indices selected 16 cmH2O. The oxygenation and saturation surrogates selected 24 cmH2O, the upper tested value. With overdistension penalty weights of 1.0, 1.65, and 2.5, the regional-balance target remained 16 cmH2O. These are results from one seeded computational scenario, not estimates of optimal PEEP for a child.

Exploratory one-at-a-time analyses held the primary scenario and seed fixed while varying selected assumptions over illustrative ranges (Appendices). Changing mean opening pressure from 8 to 12 cmH2O shifted the regional-balance target from 14 to 18 cmH2O. Changing the overdistension-threshold shift from 9.5 to 13.5 cmH2O moved that target from 14 to 16 cmH2O. Limited changes in the hysteresis gap, derecruitment rate, and dependent perfusion or ventilation weights did not move the selected regional target in this scenario, although some altered the matched-pressure memory effect. The oxygenation surrogate selected 24 cmH2O throughout these runs because of its specified monotonic form. A post hoc alternative weighting of the gas-exchange composite selected 16 to 18 cmH2O. These ranges are illustrative, not measured parameter uncertainty or evidence of broad robustness.

Illustrative output

Reproducibility and Access

The PRE v0.7 source code and original outputs are publicly available at https://github.com/taffache-hash/pediatric-recruitment-engine-pre. The engine used here is unchanged from commit 7103a11; the archived release is available on Zenodo [9]. After installing the listed dependencies, running code/run_pre_v07.py regenerates the original numerical tables and figures within a specified software environment. The revised Supplementary Methods S1, its parameter and check tables, and the separate exploratory sensitivity script and CSV accompany this manuscript revision (Appendices). The new sensitivity analysis is not part of the frozen v0.7 release.

## Discussion

The principal contribution of PRE is not the identification of a physiologic relationship, a patient-specific response, or a clinically appropriate positive end-expiratory pressure (PEEP). Rather, it is a transparent and reproducible teaching model that makes explicit how a defined set of assumptions can generate divergent outputs during a simulated PEEP trial. In the worked scenario, the oxygenation surrogate favored the highest tested PEEP, whereas several composite and clearance-related indices favored an intermediate pressure. This divergence should not be interpreted as evidence for a clinical target. It follows from the equations, weights, and penalty functions selected for the model and is intended to illustrate why no single output should be treated as a sufficient representation of the effects of PEEP.

PRE belongs broadly to a long-standing family of computational approaches that represent lung heterogeneity through distributions of opening and closing pressures and, in some models, recruitment and derecruitment kinetics. These approaches have been used to examine how heterogeneous unit behavior can shape respiratory-system mechanics and pressure-volume behavior. For example, a recruitment-derecruitment model fitted to experimental mouse data reproduced changes in lung mechanics during acute lung injury [10]. Such work provides biologic and experimental context for using heterogeneous-unit representations, but it does not validate the parameter values, update rules, or output equations used in PRE.

Other computational approaches are substantially closer to clinical application because they are fitted to patient measurements. In adult acute respiratory distress syndrome (ARDS), model-based methods have used pressure-volume measurements and recruitment-related variables to estimate patient-specific behavior during PEEP trials [11]. Virtual-patient approaches have also been evaluated against clinical measurements to predict mechanical consequences of changes in PEEP [12]. These models differ fundamentally from PRE: they use patient data for parameter estimation or prediction and require explicit evaluation against observed clinical variables. PRE does not ingest bedside data, estimate an individual patient's physiology, or predict measured oxygenation, carbon dioxide tension, respiratory mechanics, regional ventilation, or outcome. It should therefore not be described as a virtual patient, a patient-specific model, a digital twin, or a PEEP decision-support system.

At a further level of complexity, multiscale computational models have incorporated airway and alveolar geometry, tissue mechanics, surfactant behavior, and time-dependent recruitment-derecruitment dynamics to investigate mechanisms of ventilator-induced lung injury [13]. These models may improve mechanistic representation, but their sophistication does not by itself establish bedside validity or clinical utility. PRE deliberately takes a different approach: it reduces biological detail so that the relationship between assumptions and outputs remains inspectable by learners and instructors. Its four-by-four regional display is consequently a conceptual visualization and not an electrical impedance tomography (EIT) reconstruction or a representation of measured regional strain.

The clinical literature reinforces the need for this distinction. In children with acute lung injury, EIT-guided PEEP titration has been studied using real-time regional compliance analysis and measured changes in collapse and overdistension [7]. This is an important clinical-monitoring approach, but it is not equivalent to a computational teaching model. PRE can be used to discuss the conceptual trade-off that EIT aims to observe, while it cannot reproduce, substitute for, or validate EIT-derived measurements. More generally, current pediatric literature recognizes that the evidence supporting PEEP titration strategies beyond conventional approaches remains limited; methods based on compliance, imaging, esophageal pressure, or EIT require further pediatric outcome data [14].

The same caution applies to emerging physiological-model and digital-twin decision-support systems. An adult randomized trial of advice generated by a physiological-model/digital-twin system did not demonstrate improvement in its primary endpoint of driving pressure, despite selected secondary physiological findings [15]. This illustrates that linking a model to patient data and deploying it in clinical care are distinct steps, each requiring prospective assessment. PRE has not reached either step and makes no claim to do so.

Accordingly, PRE is best viewed as a supervised aid to model literacy: learners can identify assumptions, examine directionality, and see dependencies among outputs. Fixed seeds, available code, numerical reruns, and scripted checks make the implementation inspectable. The checks are limited to execution and expected model behavior, while the exploratory sensitivity results show that some target locations depend on assigned values. These activities do not establish physiologic accuracy, parameter calibration, broad robustness, generalizability, educational benefit, or clinical safety.

Future work should preserve the separation between educational, mechanistic, and clinical objectives. A clinically oriented model would require prospectively specified target variables, pediatric datasets acquired during defined PEEP trials, parameter calibration, evaluation in independent cohorts, uncertainty analysis, and assessment of whether model use improves decisions or outcomes without harmful overconfidence. An educational evaluation would require different methods, including assessment of conceptual understanding, calibration of learner confidence, and potential negative educational transfer. Until such studies are completed, PRE should remain an open, assumption-inspection model and must not guide ventilator settings.

## Conclusions

PRE v0.7 is a transparent computational model for inspecting assumptions about pediatric lung recruitment during a simulated PEEP sequence. Its frozen code reproduces archived numerical outputs and passes 10 script-defined execution and expected-behavior checks. Selected outputs vary under some illustrative parameter changes, and no clinical or physiologic validation has been performed. The reported pressures and surrogate indices are model examples, not patient estimates. PRE may support supervised discussion of assumptions and trade-offs, but it must not guide ventilator settings.

## Appendices

Full equations, sampling distributions, coefficient values, regional definitions, target-selection rules, and assumptions

https://docs.google.com/document/d/1NBaNkHblSYnMRAX-sxx_2weMg0bFP5KSYKxOUOE47ho
